# Magnitude and determinants of alcohol use disorder among adult population in East Asian countries: A systematic review and meta-analysis

**DOI:** 10.3389/fpubh.2023.1144012

**Published:** 2023-02-28

**Authors:** Getaneh Mulualem Belay, Katherine Ka Wai Lam, Qi Liu, Cynthia Sau Ting Wu, Yim Wah Mak, Ka Yan Ho

**Affiliations:** School of Nursing, Hong Kong Polytechnic University, Hong Kong, Hong Kong SAR, China

**Keywords:** alcohol use disorder, determinants, prevalence, East Asian countries, systematic review and meta-analysis

## Abstract

**Introduction:**

Alcohol use disorder is a medical condition characterized by an impaired ability to control or stop alcohol use despite adverse health outcomes. Despite several studies that have analyzed the prevalence and determinants, their results have been equivocal, and the reasons for the differences in prevalence rates and determinants of AUD across nationalities are unknown. Hence, this study estimated the pooled prevalence of alcohol use disorder and its determinant among adults in East Asian countries.

**Methods:**

Articles were searched from PubMed, Web of Science, EMBASE, PsycINFO, and Scopus. All observational study designs that fulfilled the predefined criteria were included in the study. The findings were reported following the Preferred Reporting Items for Systematic Reviews and Meta-Analysis (PRISMA). The quality and heterogeneity of articles were assessed using the new castle-Ottawa scale (NOS) and I^2^, respectively. Additionally, publication bias was checked through funnel plot and Egger's regression test.

**Results:**

A total of 14 articles with 93, 161 study participants were considered in the study. Of which 9 studies were included in the meta-analysis of the 1-year prevalence of alcohol use disorder, 6 in the lifetime, 9 in alcohol abuse, and 8 in alcohol dependency. Consequently, the overall pooled prevalence of one-year alcohol use disorder was 8.88% (95% CI: 6.32, 11.44), lifetime 13.41% (95%CI: 8.48, 18.34), alcohol abuse 5.4% (95% CI: 2.66, 8.13), and alcohol dependency 4.47% (95% CI: 2.66, 6.27). In the subgroup analysis by country, the highest 1-year and lifetime pooled prevalence of alcohol use disorder was observed in Korea at 9.78% (95% CI:4.40, 15.15) and 16.73% (95% CI: 15.31, 18.16), respectively. Besides, smoking (OR: 3.99; 95% CI: 1.65, 6.33) and male gender (OR: 5.9; 95% CI: 3.3, 8.51) were significant determinants of alcohol use disorder.

**Conclusions:**

The magnitude of alcohol use disorder was high among adults in East Asian countries. Smoking and male gender were the key determinants of alcohol use disorders.

## Introduction

Alcohol use disorder (AUD) is a medical condition characterized by an impaired ability to control or stop alcohol use despite adverse health outcomes (1). According to the Diagnostic and Statistical Manual of Mental Disorders (DSM) and International Classification of Disease (ICD), AUD is defined as continued alcohol use despite negative biological, psychological, behavioral, and social consequences within the last 12 months. In the DSM-4, alcohol abuse and alcohol dependence were defined separately, while DSM-5 integrates these two disorders into a single disorder called AUD (1).

According to a national survey on drug use and health, about 71.4% American people aged >18 years were struggling with alcohol use, while 10.2% of American people aged ≥12 years had AUD (2). In Asia, about 4.9 million (37.2%) people aged ≥26 years were alcohol users in 2020, of whom 993,000 (7.5%) had AUD. Approximately 32.2% (656,000) of young adults (aged 18–25 years) were using alcohol, and 9% of them (184,000) had developed AUD (3). In recent years, the proportion of heavy and binge drinkers among the adult populations of many Asia-pacific countries and territories has increased and is now seen in approximately one in every two men and one in every five women (4). Hence, AUD is a major health problem in Asia (5).

According to a 2022 report from the World Health Organization (WHO), approximately 200 health conditions and injuries are associated with the harmful use of alcohol, including road injuries, violence, suicide attempts, and medical problems such as liver disease, cardiovascular disease, cancer, tuberculosis, and HIV/AIDS (6). The mortality attributable to alcohol consumption was much higher than that related to tuberculosis, HIV/AIDS, and diabetes (7). Globally, approximately 3 million deaths result from the harmful use of alcohol per year. For instance, in 2016, approximately 2.3 and 0.7 million men and women died due to AUD, respectively (7). In particular, an estimated 106.5 and 26.1 million Disability-adjusted life years (DALYs) in men and women were attributed to alcohol use, respectively (8). Generally, AUD results in diminished brain function, stomach complications, liver and kidney failure, and then finally leads to death (9). Besides, high-risk drinking causes societal-related health problems such as the breakdown of social relationships, domestic violence, compromised family care, mental health problems (e.g., depression, anxiety, and related forms of substance misuse), unsafe sex, financial problems, and homelessness (10).

The development of AUD is associated with multiple societal factors, including the availability of alcohol (11), level of economic development (12), and culture (12), as well as with individual factors like age (13, 14), gender (13–15), family history of alcohol use and smoking status (14, 15).

To tackle AUD, the WHO proposed a global strategy for reducing alcohol consumption that has been a public health priority since 2010. Pharmacological and psychosocial management approaches (e.g., brief interventions, cognitive behavioral therapy, acceptance and commitment therapy, and 12-step programs) have been used for treating AUD (16). However, AUD remains a major issue among the adult populations of East Asian countries.

Several studies have analyzed the prevalence and determinants of AUD in East Asian countries, but their results have been equivocal. In addition, the reasons for the differences in prevalence rates and determinants of AUD across nationalities are unknown. Therefore, a systematic review and meta-analysis is required.

The current study aimed to estimate the prevalence of AUD, and to identify its determinants, through the analysis of a large sample of participants. Large-pooled samples provide more precise estimates than individual studies. This study provides timely and comprehensive data for governments and policymakers and could inform the design of new prevention and treatment strategies for people with AUD in East Asian countries.

## Methods

### Searching strategies and data sources

The study protocol was registered in the International Prospective Register of Systematic Reviews and Meta-Analysis (PROSPERO; registration number: CRD42022361994). The Preferred Reporting Items for Systematic Review and Meta-Analysis (PRISMA) guidelines were followed when reporting the results of this study (17) (Supplementary material 1). All potentially relevant articles were retrieved systematically from electronic databases including PubMed, EMBASE, Web of Science, PsycINFO, and Scopus. The search was limited to studies conducted in East Asian countries. The identified articles were exported into citation management software (EndNote version 20; Clarivate, London, UK) for further screening and assessment. The databases were searched using the following terms: “Alcohol abuse” OR “Alcohol depend^*^” OR “Alcohol use disord^*^” OR “Alcohol consumption” OR “Alcohol addiction” OR “alcohol intoxication” AND “prevalence” OR “incidence” OR “proportion” OR “magnitude” OR “burden” AND “determinants” OR “reasons” OR “factors” OR “associated factors” OR “risk factors” OR “facto^*^” OR “predictors” AND “Adults” OR “young adults” OR “adulthood” OR “adolescents” OR “young adult populations” AND “China” OR “Hong Kong” OR “Japan” OR “Taiwan” OR “South Korea” OR “North Korea” OR “Macau” OR “Mongolia.” Searches were conducted from September 10 to October 5, 2022.

### Inclusion and exclusion criteria

Articles were selected and screened on the basis of predefined eligibility criteria. All observational studies, including but not limited to case-control, cross-sectional, and cohort studies, reporting the prevalence and/or magnitude of AUD and/or its determinants among adults in East Asian countries were included in this review. The search was limited to articles published in English since 2005. Systematic reviews, editorials, trials, qualitative studies, and conference papers were excluded, as were articles without full text (established through communication with the primary authors of the articles). We also excluded studies of adults with psychiatric or other health problems. This was because mental health issues increase the severity of AUD (18–20), and people with medical conditions are less likely to use alcohol because it exacerbates symptoms and is often contraindicated (21).

### Outcome measures

The outcome measures of the current study included the prevalence of AUD and its determinants. In the DSM-IV and DSM-5, AUD is defined as the presence of at least 2 of the following 11 behaviors/symptoms: (I) had times when you ended up drinking more, or for longer, than you intended; (II) wanted to cut down or stop drinking more than once, but could not; (III) spent considerable time drinking, being sick or getting over other aftereffects of drinking; (IV) wanted to drink so badly that you could not think of anything else; (V) being sick from drinking often interfered with taking care of your family or home, or caused problems at school; (VI) continued to drink even though it caused trouble with your family or friends; (VII) gave up or cut back on activities that were important or interesting to you, or put pressure on you to drink; (VIII) entered into situations more than once, while or after drinking, which increased your chances of getting hurt; (IX) continued to drink even though it was making you feel depressed or anxious; (X) had to drink much more than you once did to achieve the effect that you wanted or found that your usual number of drinks had much less effect than before; or (XI) found that, when the effects of alcohol were wearing off, you had withdrawal systems like trouble sleeping, shakiness, restlessness, nausea, sweating, a racing heart or seizure (1).

In the DSM-5, AUD encompasses alcohol abuse and dependence. Alcohol abuse is defined as the continuation of drinking despite recurrent social, interpersonal, health and legal problems caused by alcohol use (22), or by the presence of at least one of the following symptoms: (I) sickness caused by drinking often interfered with taking care of your home or family; (II) entered into a situation more than once, during or after drinking, which increased your chances of getting hurt (e.g., swimming, driving, unsafe sex, or walking in a dangerous area); (III) arrested or held at the police station more than once, or experienced other legal problems, because of drinking; or (IV) continued drinking despite it causing trouble with your family or friends (1).

Alcohol dependence refers to the need for repeated consumption of alcohol or impaired control over drinking (23). According to the DSM-IV, the presence of three of the following symptoms indicates alcohol dependence: (I) spend a lot of time drinking; (II) wanted to cut down or stop drinking more than once but could not; (III) had to drink more than you once did to achieve the effect that you want; (IV) experience withdrawal symptoms; (V) had times when you ended up drinking more, or for longer, than you intended; or (IV) continued drinking despite it making you feel depressed or anxious (1).

### Study screening and selection

All articles retrieved from the electronic databases were imported into EndNote, and duplicates were identified and removed. Then, five authors (GMB, KYH, KKWL, CSTW, and QL) screened and reviewed the titles and abstracts of each article. Disagreements were resolved through discussion and repeating the review process if necessary. Finally, the authors reviewed the full texts of the articles selected on the basis of their titles and abstracts.

### Data extraction

Data extraction was done using Microsoft Excel (Microsoft Corp., Redmond, WAQ, USA) following the guidelines of Joanna Briggs Institute. The following information was systematically reviewed and extracted by two authors (GMB and KYH): first author, year of publication, country, study area, study population, study design, sample size, prevalence of alcohol abuse, alcohol dependence, and AUD (one year and lifetime), and adjusted odds ratios (AORs) of factors significantly associated with AUD (with 95% confidence intervals [CIs]). Discrepancies were resolved by repeating the data extraction procedure, as well as through discussion with a senior member of the research team (YWM). If additional information was needed, the primary author of the articles was contacted by email.

### Quality appraisal

The Newcastle-Ottawa Scale (NOS) risk of bias tool was used for assessing cross-sectional, case-control, and cohort studies (24, 25). The NOS has three components (selection, comparability, and outcome/exposure) and has been validated for application to cross-sectional (rated from 0–10 stars), cohort and case-control studies (rated from 0–9 stars). For the selection component, star ratings of 0–5 are provided for cross-sectional studies, and 0–4 for case-control and cohort studies. For the comparability and outcome/exposure components, star ratings of 0–2 are provided, while study designs are rated as 0–3. Articles with 3 or 4 stars for the selection component, 1 or 2 stars for the comparability component, and 2 or 3 stars for the outcome/exposure component were categorized as “good quality.” Articles receiving 2 stars for the selection component, 1 or 2 stars for the comparability component, and 2 or 3 stars for the outcome/exposure component were classified as “fair quality.” Articles having 0 or 1 star for the selection component, 0 stars for the comparability component, or 0 or 1 star for the outcome/exposure component were classified as “poor quality” (26). The quality of the included studies was systematically and independently appraised by the authors (GMB and KYH) of this study. Disagreements among the two authors were resolved by repeating the quality appraisal procedure, and through discussion with a senior research team member (YWM).

### Data synthesis

A narrative description approach was used to present the results of articles that did not report the outcomes of interest. All other articles were included in the meta-analysis, and STATA software (version 14; Stata Corp., College Station, TX, USA) was employed to calculate the overall prevalence rates of AUD, alcohol abuse and alcohol dependency. Pooled ORs were calculated for the determinants of the outcomes of interest. To minimize heterogeneity, a random effects model was employed (27). *P*-values < 0.05 were considered statistically significant.

### Publication bias and heterogeneity

Heterogeneity among the included studies was assessed by visual inspection of the funnel plot and Egger's regression test. Heterogeneity was considered low, moderate, and high when the I^2^ values were <25%, 25–75%, and >75%, respectively (28).

## Results

### Search results

**Figure 1:**
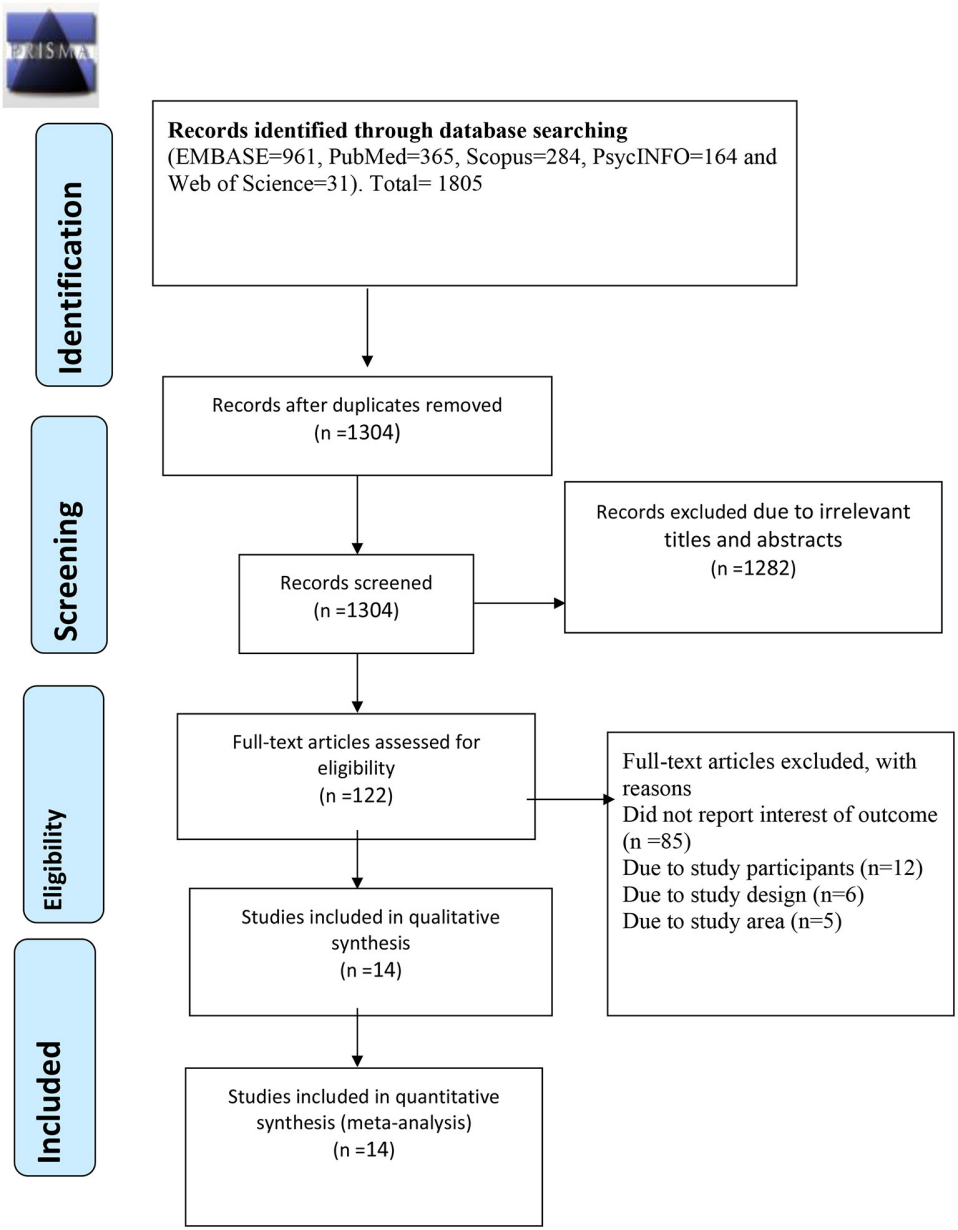
A flow diagram of the process of article identification and selection.

A total of 1,805 articles were retrieved (EMBASE, *p* = 961; PubMed, *p* = 365; Scopus, *p* = 284; PsycINFO, *p* = 164; Web of Science, *p* = 31; Figure 1). After removing 501 duplicate articles, the titles, and abstracts of 1,304 articles were screened. In total, 1,282 articles were excluded because their titles and abstracts indicate that they were not relevant to this study. The remaining 122 articles were downloaded, and full-text review was conducted by the two reviewers (GMB and KYH). A further 107 articles were then excluded for various reasons, including failure to report the outcomes of interest (*p* = 85), and unsuitable study populations (*p* = 12), study designs (*p* = 6), and study areas (*p* = 5). Of the excluded articles, one article (29) was not considered in the study as it was done through a meta-analysis which did not meet our inclusion criteria of observational studies. And, the other one (30) did not report the 1-year and lifetime prevalence of AUD, but rather reported the co-occurrence of mental disorders and AUD in past 30 days. As per our inclusion criteria, we excluded studies of adults with psychiatric or other mental health problems. Therefore, 14 peer-reviewed articles (31–44) were included in this systematic review and meta-analysis; 9 of them were suitable for analysis of the 1-year prevalence of AUD, 6 for analysis of the lifetime prevalence of AUD, 9 for analysis of the prevalence of alcohol abuse, and 8 for analysis of the prevalence of alcohol dependence.

### Characteristics of the included studies

The 14 articles in this systematic review and meta-analysis included 93,161 participants (Table 1). The sample size of the included studies ranged from 258 to 36,157 participants, and they were published from 2005 to 2021. Most of the studies (32–36, 38–44) were cross-sectional, although there was one case-control (31), one cohort (37), and one comparative cross-sectional study (38). Most of the included studies were from China (31, 32, 35, 39, 42–44), followed by Korea (33, 38, 41), Japan (34, 40), Taiwan (37), and Hong Kong (36). The highest and lowest prevalence rates of AUD were reported in China, at 16.2% (32) and 1.05%, respectively (42). The highest and lowest lifetime prevalence rates of AUD were also reported in China, at 17.7 and 3.03%, respectively (42, 44).

**Table 1:** Characteristics of included articles in this systematic review and meta-analysis (*n* = 14).

**S. No**	**References**	**Study country**	**Study area**	**Study design**	**Study population**	**Sample size**	**Diagnostic criteria**	**Alcohol abuse**	**Alcohol dependence**	**AUD (1 year)**	**Lifetime AUD**
1	Chen et al. (31)	China	Institution based	Case-control	Adults	Control: 129 Cases: 129	DSM IV	NA	NA	NA	NA
2	Lee et al. (38)	Korea	Community based	Comparative cross sectional	Adults	7,867	DSM-IV	2	5.1	7.1	17.2
3	Kim et al. (36)	Hong Kong	Community based	Cross-sectional	Adults	9,860	DSM-IV	6.7	3	9.7	NR
4	Zhou et al. (44)	China	Community based	Cross sectional	Adults	9,866	DSM-IV	2.1	6.6	8.7	17.7
5	Guo et al. (32)	China	Community based	Cross sectional survey	adults	3,171	DSM-IV	2.7	13.5	16.2	NR
6	Hahm et al. (33)	Korea	Community based	Cross sectional survey	Adults	1,059	DSM-1IV	2.5	5	7.5	15.6
7	Orui et al. (40)	Japan	Community based	Cross sectional survey	Adults	1,684	DSM-IV	9.1	1.1	10.2	NR
8	Zhang et al. (42)	China	Community based	Cross sectional survey	Adults	4,528	DSM-5	NR	NR	1.05	3.03
9	Lee et al. (39)	China	Community based	Cross sectional survey	Adults	5,201	DSM-IV	4.7	1	NR	5.7
10	Ishikawa et al. (34)	Japan	Community based	Cross sectional survey	Adults	4,130	DSM-IV	7.3	0.9	NR	8.2
11	Ji et al. (35)	China	Community based	Cross sectional survey	Adults	36,157	DSM-1V	11.56	NR	NR	NR
12	Lee et al. (37)	Taiwan	Community based	Cohort	Adults	979	DSM-IV	NR	NR	1.75	NR
13	Park et al. (41)	Korea	Community based	Cross sectional survey	Adults	6,276	DSM-IV	NR	NR	14.7	NR
14	Zhi et al. (43)	China	Community based	Cross sectional survey	Adults	2,129	DSM-IV	NR	NR	4.16	16.17

NA, Not applicable; NR, Not reported.

### Quality appraisal

The NOS was used to assess the quality of the included studies. The quality ratings ranged from 6 to 8 stars for cross-sectional studies (32–36, 38–44), seven for case-control studies (31) and six for cohort studies (37). The most common problems were an unsatisfactory response rate, failure to compare respondents and non-respondents (38, 41), and a lack of CIs for the determinants of AUD (32, 37).

### Narrative description

Five studies (31, 34, 35, 39, 42) were not considered in the analysis of the 1-year prevalence of AUD: four of them (31, 34, 35, 39) did not report the prevalence rate, while the remaining study, which was conducted in China (42), reported it on the basis of the DSM-5 (which has different diagnostic criteria to the DSM-IV).

Eight articles (31, 32, 35–37, 40–42) were not included in the analysis of lifetime prevalence of AUD because they did not report that outcome. Four of those studies were conducted in China, including one case-control study (31) and three cross-sectional studies (32, 35, 42); the remaining four studies were from Korea (41), Hong Kong (36), Taiwan (37), and Japan (40).

Nine articles (32, 34, 35, 37, 39, 40, 42–44) were excluded from the analysis of determinants of AUD: six of them did not report these data (34, 35, 39, 40, 42, 43) and two did not provide 95% CIs allowing computation of the standard error of the log odds ratio (32, 37). A further study reported that unmarried marital status was a significant factor for AUD; however, because no other studies reported on marital status, this study was not included in the analysis (44). Therefore, five articles were included in the analysis of determinants of AUD, one of which was a case-control study conducted in China involving 129 controls and 129 cases; that study reported that history of family alcohol use (aOR: 4.4; 95% CI: 2.94, 658), smoking (aOR: 3.39; 95% CI: 1.48, 7.77), higher education level (aOR: 0.88; 95% CI: 0.78, 0.99), and induced drinking during childhood (aOR: 6.09; 95% CI:2.56, 14.51) were determinants of AUD (31).

### One-year pooled prevalence of AUD

**Figure 2:**
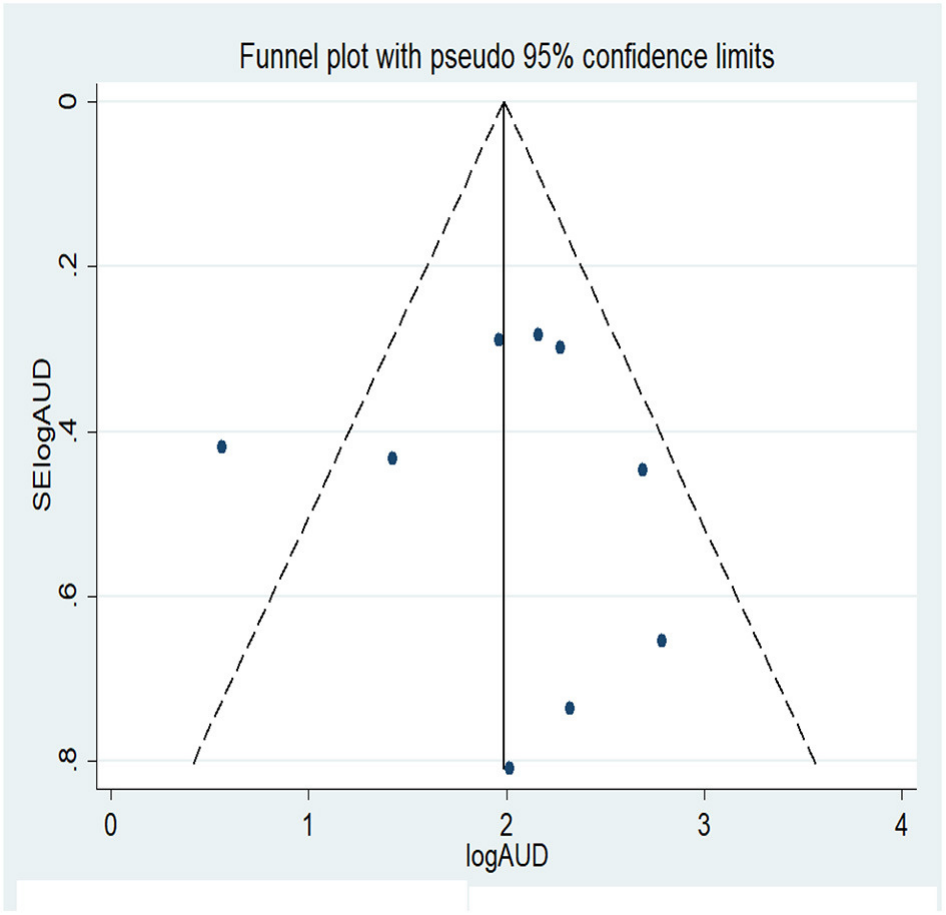
Funnel plot of publication bias for 1 year prevalence of AUD.

**Figure 3:**
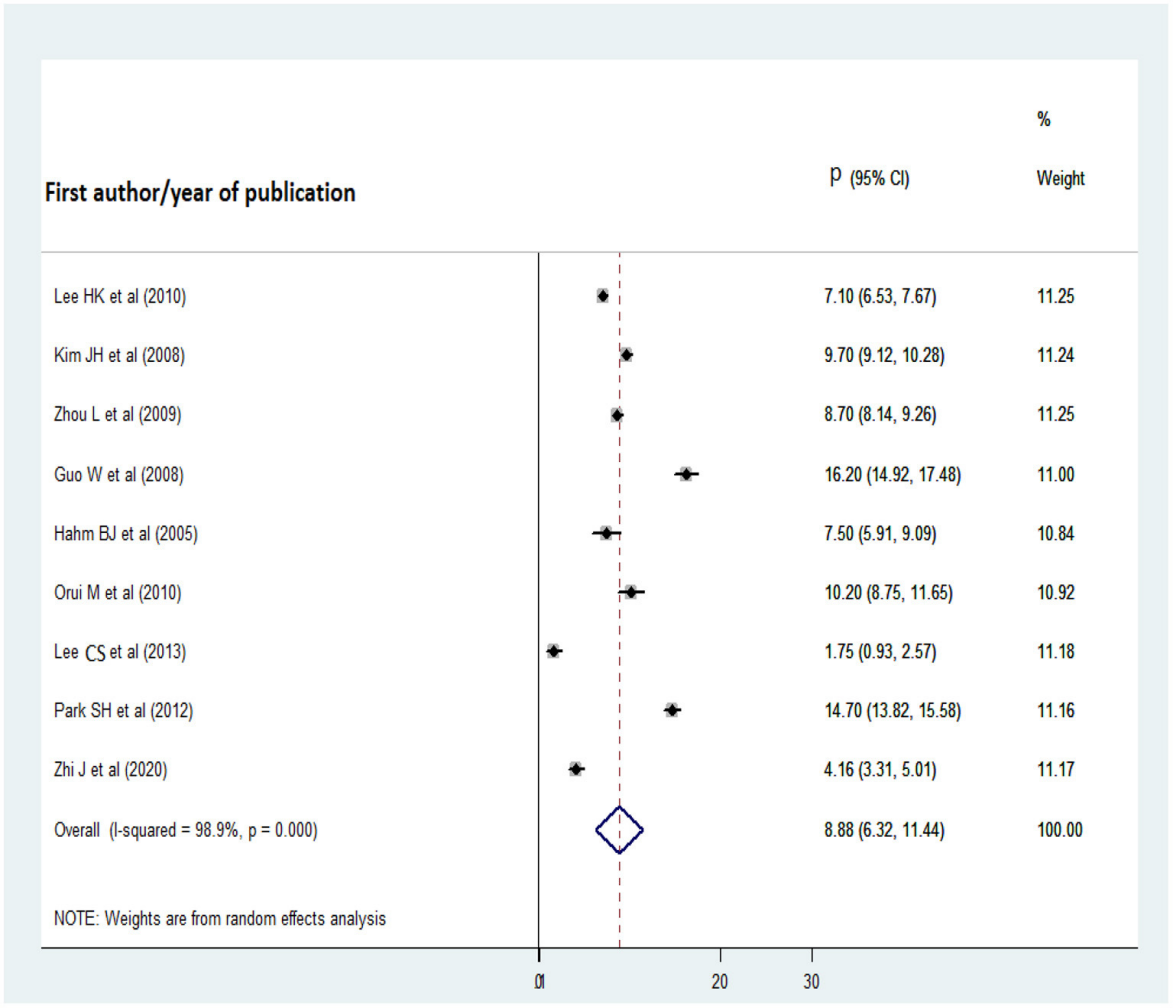
Forest plot of the 1-year pooled prevalence of AUD.

Nine studies were included in the analysis of the 1-year pooled prevalence of AUD. Before computing the pooled prevalence, publication bias was assessed with a funnel plot and Egger's regression test. Visual inspection of the funnel plot revealed a symmetrical distribution of the studies and the Egger's regression test statistic was non-significant (t = 0.04, *p* = 0.970); therefore, there was no significant publication bias (Figure 2). On the basis of the aforementioned results, nine studies were included in the analysis of the 1-year pooled prevalence of AUD, published from 2008 to 2020. The highest and lowest prevalence rates of AUD were reported in studies from China (16.2%) and Taiwan (1.75%), respectively, and the 1-year pooled prevalence of AUD disorder was 8.88% (95% CI: 6.32, 11.44) (Figure 3).

A subgroup analysis was conducted for Korea and China because at least two studies conducted in these countries reported the 1-year pooled prevalence of AUD disorder (Korea, 9.78%; 95% CI: 4.40, 15.15; China, 9.66%; 95% CI: 4.31, 15.01).

### Pooled lifetime prevalence of AUD

**Figure 4:**
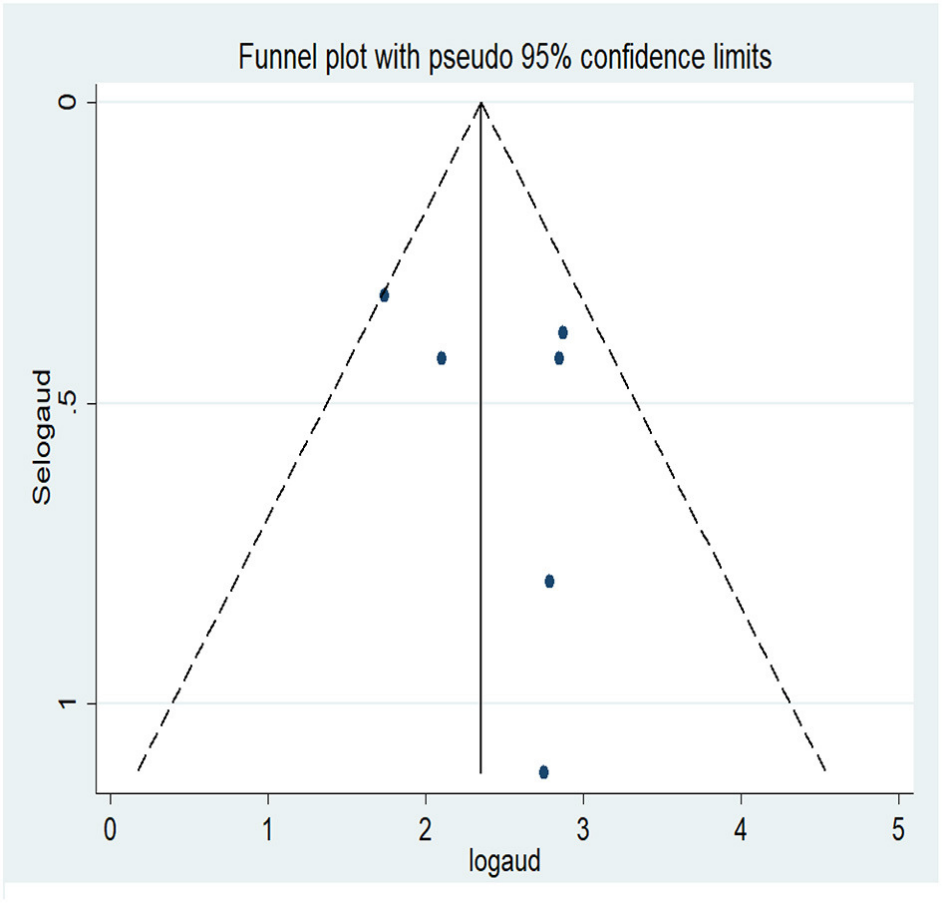
Funnel plot of publication bias for lifetime prevalence of AUD.

**Figure 5:**
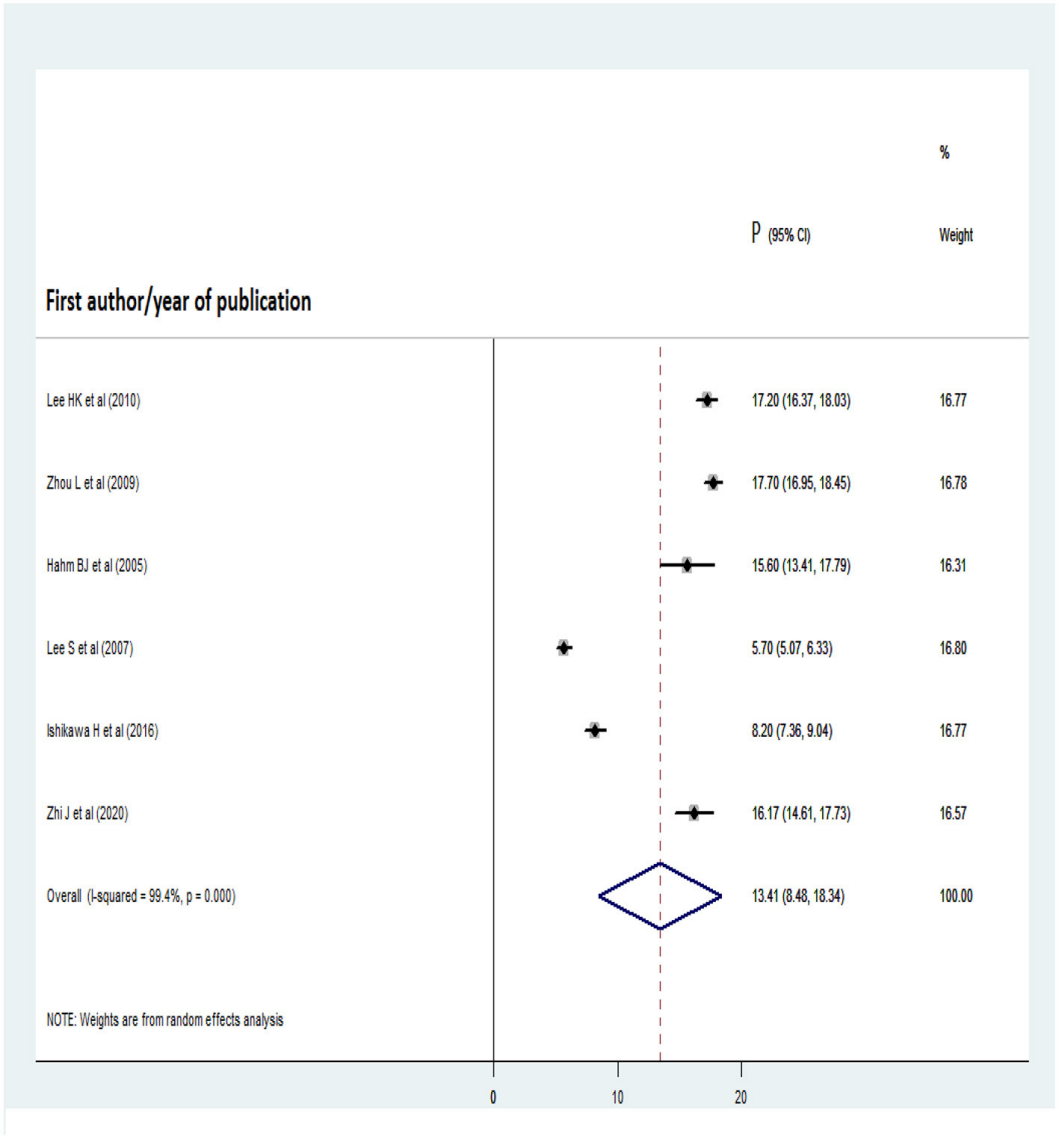
Forest plot for lifetime pooled prevalence of AUD.

The lifetime pooled prevalence of AUD was estimated on the basis of six studies from Korea, China, Hong Kong, and Japan (total of 30,252 participants). Prior to the analysis, publication bias was assessed with a funnel plot and Egger's regression test. The symmetrical distribution of the articles in the funnel plot indicated that there was no publication bias (Figure 4), as did the non-significant Egger's regression test statistic (t = 0.88, *p* = 0.427). The overall pooled lifetime prevalence of AUD was 13.41% (95% CI: 8.48, 18.34) (Figure 5). In a subgroup analysis by country, the pooled lifetime prevalence was highest for Korea, at 16.73% (95% CI: 15.31, 18.16), followed by China (13.18%; 95% CI: 4.34, 22.02).

### Pooled prevalence of alcohol abuse and dependence

**Figure 6:**
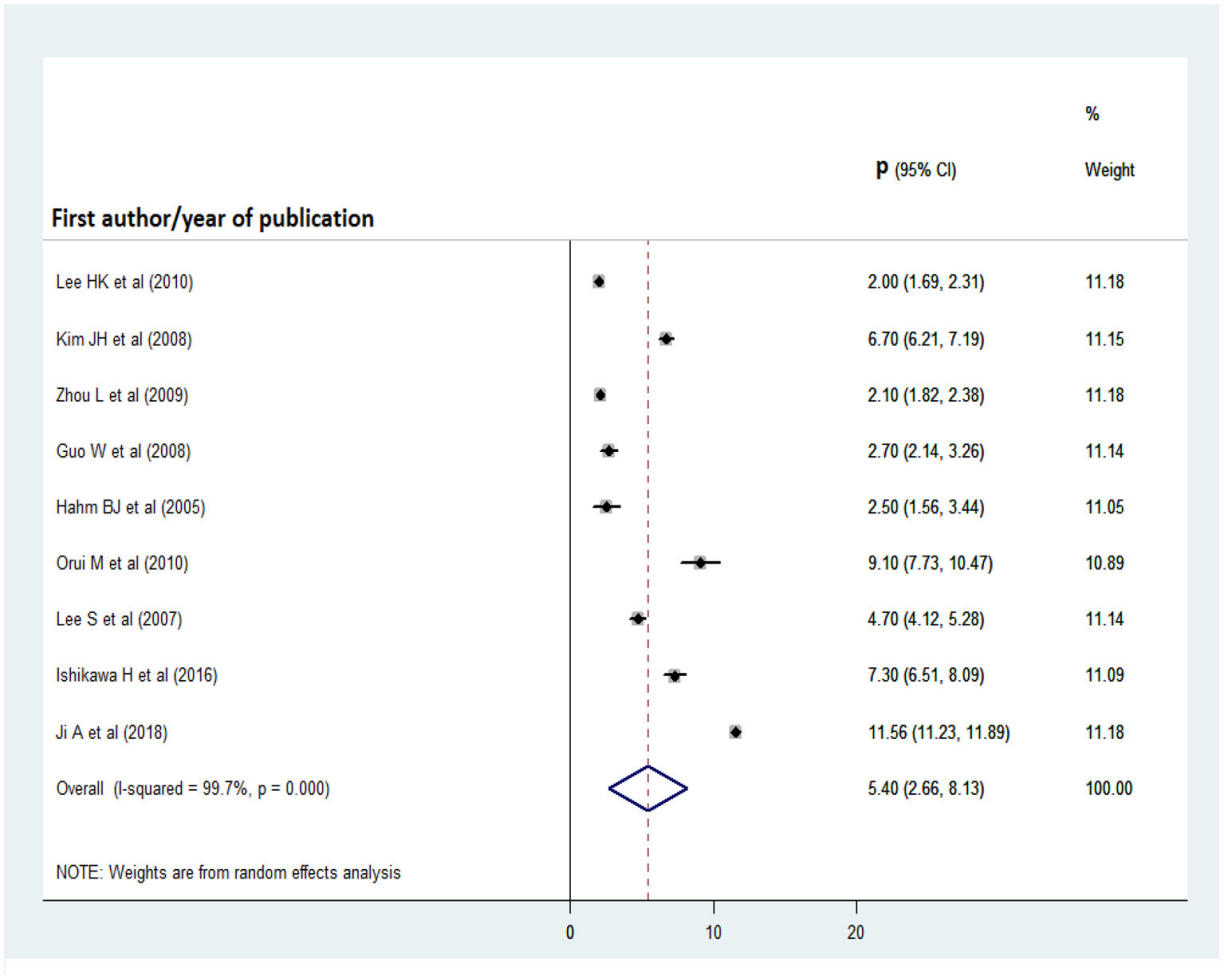
Forest plot for the pooled prevalence of alcohol abuse.

**Figure 7:**
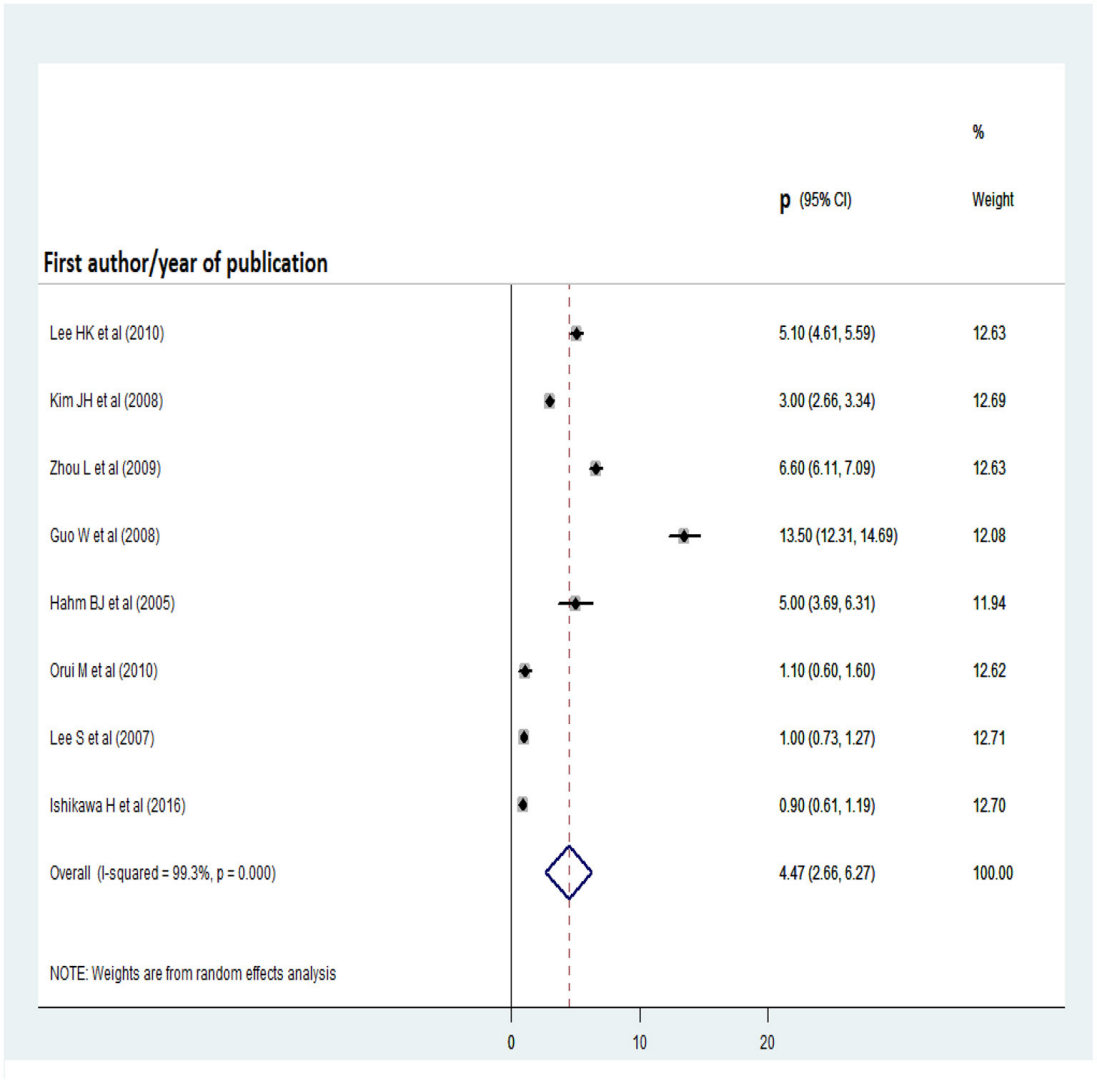
Forest plot for pooled prevalence of alcohol dependency.

As shown in Figures 6, 7, nine and eight articles were included in the analyses of alcohol abuse and dependence, respectively. Before the analyses, publication bias among studies was checked for by inspecting their distribution on the funnel plot and performing Egger's regression test. There was no significant publication bias among the studies of alcohol abuse or dependence, as evidenced by the Egger's regression test statistics (t = −2.17, *p* = 0.067 and t = 1.96, *p* = 0.098, respectively). The prevalence rate of alcohol abuse was highest in China (11.56%) and lowest in Korea (2%), while that of alcohol dependence was highest in China (13.5%) and lowest in Japan (0.9%). The overall pooled prevalence of alcohol abuse was 5.4% (95% CI: 2.66, 8.13), while that of alcohol dependence was 4.47% (95% CI: 2.66, 6.27).

### Determinants of AUD

Among the studies included in the analysis of determinants of AUD, statistically significant factors included male gender (38, 41), history of family alcohol use (31, 41), smoking (31, 36, 41), binge drinking (36), education level (31, 33, 36, 38, 41, 44), income (38, 44), and unmarried status (41).

**Figure 8:**
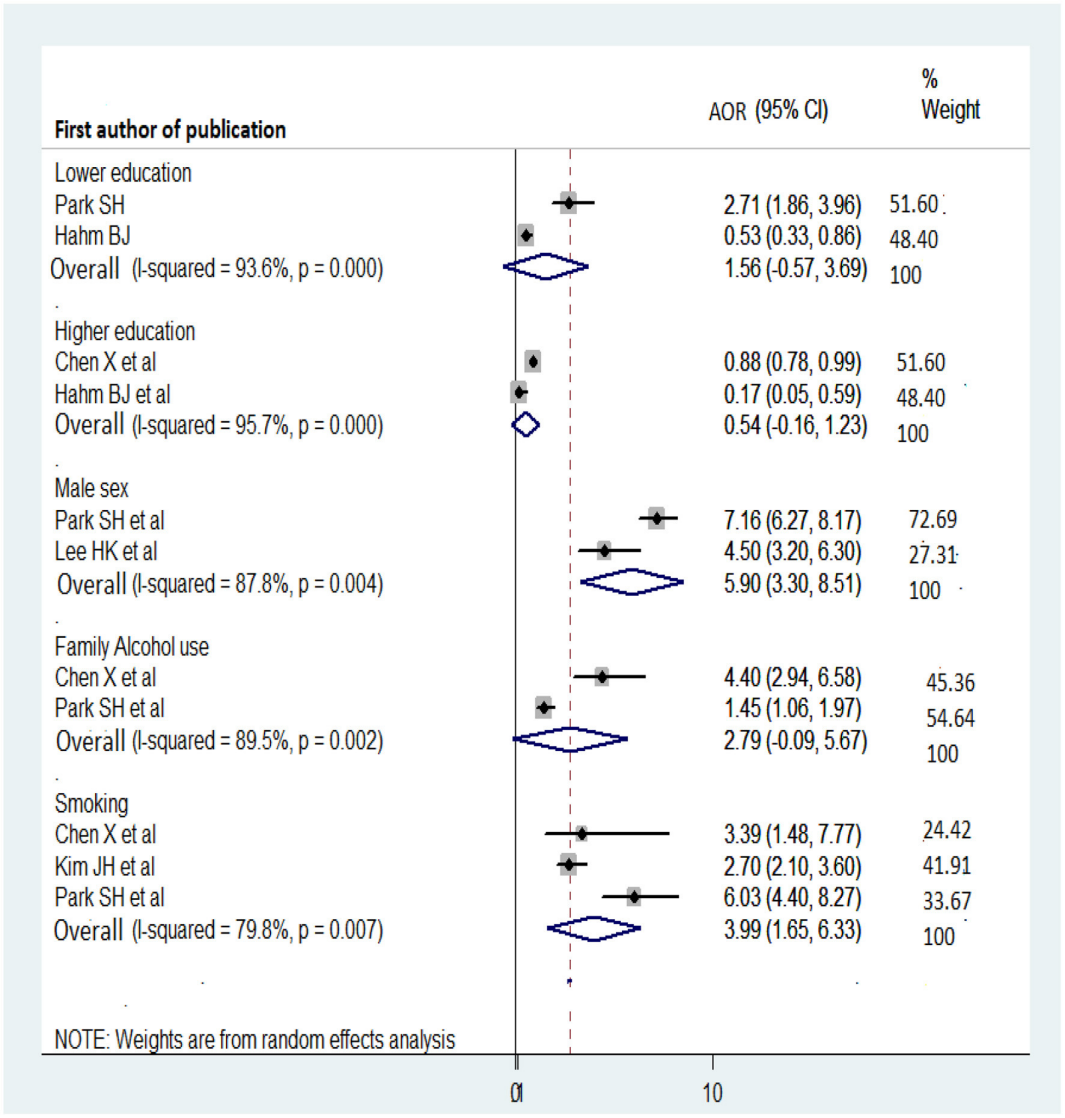
Forest plot for the determinants of AUD.

Studies conducted in China (31), Korea (41), and Hong Kong (36) revealed a positive relationship between smoking and AUD (overall OR: 3.99; 95% CI: 1.65, 6.33). History of family alcohol use was a determinant of AUD in a Chinese study (AOR: 4.4; 95% CI: 2.94, 658), as well as a Korean one (AOR: 1.45; 95% CI: 1.06, 1.97), although this factor was not significantly associated with AUD when all studies were considered (overall OR: 2.79; 95% CI: −0.09, 5.67). Sex was also identified as a determinant of AUD. Male gender was statistically significantly associated with AUD (overall OR: 5.9; 95% CI: 3.3, 8.51). Regarding education level, the results were inconsistent (31, 33, 38, 41); while some studies indicated that a higher education level was a protective factor for AUD (31, 33), others found that a lower education level was a protective factor (33). In the analysis of all studies, the overall odds ratios for lower and higher education levels were 1.68 (95% CI: 0.25, 3.12) and 0.54 (95% CI: −0.16, 1.23), respectively (Figure 8).

## Discussion

Although various management strategies are emerging, AUD remains a major problem in East Asian countries. However, the reported prevalence and determinants of AUD vary among countries. Therefore, this systematic review and meta-analysis aimed to determine the pooled prevalence of AUD among the adult populations of East Asian countries and calculated pooled odds ratios for its determinants. The pooled 1-year prevalence of AUD was high, at 8.88% (95% CI: 6.32, 11.44), as was the lifetime prevalence, at 13.41% (95% CI: 8.48, 18.34) the rates of alcohol abuse and alcohol dependence were 5.4% (95% CI: 2.66, 8.13) and 4.47% (95% CI: 2.66, 6.27), respectively.

The pooled prevalence of AUD in this study was generally lower than that reported in American at 12% (45), Australia at 18% (46), Canada 19%, and in Brazil at 18.5% (14).

The difference in prevalence of AUD between East Asian and Western countries could be explained by cultural norms and beliefs. In Mediterranean countries, alcohol is integrated into daily life and activities (wet drinking culture) (47). This accords with the results of a comparative study showing that, in cultural terms, Western countries value wine more so than China (48). Second, most young adults in Western countries consider binge drinking normal (49), and some even rely on drinking to cope with stress in their daily lives (50). Such norms and beliefs may explain the high frequency and amount of alcohol consumption in Western countries (51), which would likely lead to a higher prevalence of AUD. In 2020, the alcohol use rate among Asian people aged ≥26 years was only 37.5% (52), which was much lower than the rate reported in the US (54.6%) (2).

The pooled prevalence rates of lifetime and 1-year AUD in the current study were higher than those reported for West Asian countries, including Israel (5.9%), Iraq (0.4%) (53), Lebanon (8.7%) (54), the United Arab Emirates (0.7%) (55), Jordan (0.4%) (56), and Bahrain (1.6%) (57). These differences could be explained by religious norms. In West Asian countries, Islam, which restricts alcohol use for its adherents, is the dominant religion (58–60). Moreover, while our results were derived from a meta-analysis of studies on the prevalence of AUD, studies from West Asian countries reported data for individual countries provided by the WHO. Furthermore, the prevalence variation could be attributed by difference in methodology and data collection tools used, as questionnaires adapted according to each country's culture, norms, language, and definitions may also vary (61, 62).

We performed a subgroup analysis to explore AUD by country. The highest pooled prevalence rates for 1-year and lifetime AUD were observed in Korea, at 9.78% (95% CI: 4.40, 15.15) and 16.73% (95% CI: 15.31, 18.16), respectively. This may be because of the relative affordability of alcohol in Korea, and its strong drinking culture, particularly in the context of dinners and team building activities (63). A qualitative study conducted in Hong Kong revealed that household and parental drinking were associated with increased alcohol consumption (64). According to a WHO report, alcohol use is high in Korea at 9.6 alcohol per capita (15+ liters of pure alcohol) (65). In our meta-analysis, China had the second-highest 1-year AUD prevalence, at 9.66% (95% CI: 4.31, 15.01), and the second-highest lifetime AUD prevalence, at 13.18% (95% CI: 4.34, 22.02). These rates are much higher than those reported in mainland China, possibly because alcohol has become more accessible to the Chinese population because of reduced prices (11, 66, 67), more advertisements, less strict regulation of sales (68), and an increase in the number of outlets providing alcohol (69). Second, incomes in China have been increasing, which would likely increase both alcohol consumption and AUD rates (70); alcohol use is lower among low-income groups (71). Third, exposure to alcoholic beverages is increasing in line with the number of factories producing alcoholic beverages. For instance, a modeling study on global alcohol exposure between 1990 and 2017 revealed that, globally, adult per capita alcohol consumption increased from 5.7 L in 1990 to 7.6 L in 2017 (72). Banning alcohol advertising, and making it less available and more expensive, are potential cost-effective strategies for reducing AUD (73).

In this study, smoking (overall OR: 3.99; 95% CI: 1.65, 6.33) was the main determinant of AUD; smokers were four times more likely than non-smokers to have AUD, in line with studies conducted in the United States (74–76), Brazil (14), and India (77). The association between smoking and AUD may be attributed to the synergetic effect of the chemicals present in tobacco and alcohol. For instance, a glucocorticoid receptor activated by nicotine can alter the sensitivity of the GABAergic system to ethanol (78). In addition, nicotine potentiates the rewarding effects of ethanol (79). In fact, nicotine and alcohol can both potentiate the rewarding effects of the other substance, and nicotine use increases the likelihood of relapse among those who have quit alcohol (80).

Our systematic review and meta-analysis showed that male gender is a risk factor for AUD (overall OR: 5.9; 95% CI: 3.3, 8.51). The AUD rate among males was approximately six times higher than that among females, consistent with a previous study reporting sex differences in the prevalence of AUD (81). This sex difference is mainly attributed to variations in dopamine receptor density and gonadal steroid hormones (82). In addition, the differences in societal roles between males and females may explain the gender difference in the AUD rate. For example, in East Asian countries, females are highly engaged in housework and take a role of caregiver for their family and thus have less opportunities for alcohol use. Besides, females who are pregnant or planning to get pregnant may abstain from alcohol drinking (83, 84).

Unlike Western studies that have identified several risk factors for AUD, such as the presence of mental health problems (85, 86), stigmatization and impulsiveness (87), a religious practice, education levels (88), and anxiety sensitivity (89), few studies of AUD conducted in East Asian countries explored its determinants. Eight of the studies included herein did not report determinants of AUD, such that our analysis of these factors was limited to two or three articles. Similarly, other studies conducted in China also revealed that mood and anxiety disorder (30), comorbid mental disorders, and marital status were significantly associated with AUD (29).

This systematic review and meta-analysis provided comprehensive, up-to-date data on the overall prevalence of AUD in East Asian countries, and also shed light on its determinants. Also, most of the included studies were nationwide epidemiological studies with large sample sizes, and thus high generalizability. The cohort, case-control, and cross-sectional studies in this meta-analysis were of high quality and used the NOS.

### Limitations

As well as strengths, the current study also had some limitations. First, there was considerable publication bias among the included studies. Second, some of the studies failed to report all of the outcomes of interest. For example, one study reported on alcohol abuse but not alcohol dependence, while others reported the 1-year prevalence rate of AUD but not the lifetime prevalence (or vice versa). Five articles reported both the 1-year and lifetime AUD prevalence rates, while four reported only the former. Third, the findings may not be representative of Mongolia and Macau, because none of the included articles were conducted in those countries.

## Conclusion

This systematic review and meta-analysis revealed that 1-year and lifetime AUD rates are high in East Asian countries, and that smoking and male gender are significant risk factors for AUD. The findings of this study provide baseline information for governments and policymakers that could inform new treatment approaches for AUD. Moreover, this study shows that greater awareness of the consequences of alcohol consumption, including mortality and associated morbidities, is urgently required. Finally, this review highlighted the need to focus on smokers because of their higher likelihood of developing AUD.

## Author contributions

GB: conceptualization-equal, data curation-equal, formal analysis-equal, funding acquisition-equal, investigation-equal, methodology-equal, project administration-equal, resources-equal, software-equal, supervision-equal, validation-equal, visualization-equal, writing—original draft-equal, and writing—review and editing-equal. KL: conceptualization-equal, investigation-equal, methodology-equal, supervision-equal, validation-equal, writing—original draft-equal, and writing—review and editing-equal. QL: investigation-supporting, project administration-supporting, resources-supporting, and validation-supporting. CW: conceptualization-equal, methodology-equal, resources-equal, software-equal, and writing—review and editing-equal. YM: conceptualization-equal, methodology-equal, supervision-equal, validation-equal, writing—original draft-equal, and writing—review and editing-equal. KH: conceptualization-lead, data curation-equal, formal analysis-equal, investigation-equal, methodology-lead, project administration-equal, resources-lead, software-equal, supervision-lead, validation-equal, visualization-equal, writing—original draft-equal, and writing—review and editing-equal. All authors contributed to the article and approved the submitted version.
